# Perfusion by Arterial Spin labelling following Single dose Tadalafil In Small vessel disease (PASTIS): study protocol for a randomised controlled trial

**DOI:** 10.1186/s13063-017-1973-9

**Published:** 2017-05-22

**Authors:** Mathilde M. H. Pauls, Natasha Clarke, Sarah Trippier, Shai Betteridge, Franklyn A. Howe, Usman Khan, Christina Kruuse, Jeremy B. Madigan, Barry Moynihan, Anthony C. Pereira, Debbie Rolfe, Egill Rostrup, Caroline E. Haig, Thomas R. Barrick, Jeremy D. Isaacs, Atticus H. Hainsworth

**Affiliations:** 1 Neurosciences Research Centre, Molecular and Clinical Sciences Research Institute, St George’s University of London, Cranmer Terrace, London, SW17 0RE UK; 2 Cell Biology and Genetics Research Centre, Molecular and Clinical Sciences Research Institute, St George’s University of London, Cranmer Terrace, London, SW17 0RE UK; 3 Department of Neurology, St George’s University Hospitals NHS Foundation Trust, Blackshaw Road, London, SW17 0QT UK; 4 Stroke Clinical Research Network, St George’s University Hospitals NHS Foundation Trust, Blackshaw Road, London, SW17 0QT UK; 5 Department of Neuropsychology, St George’s University Hospitals NHS Foundation Trust, Blackshaw Road, London, SW17 0QT UK; 6 Department of Neuroradiology, St George’s University Hospitals NHS Foundation Trust, Blackshaw Road, London, SW17 0QT UK; 7 Department of Neurology, Herlev Hospital, Herlev Ringvej 75, 2730 Herlev, Denmark; 8 Beaumont Hospital, Beaumont, Dublin 9 Ireland; 9 Joint Research and Enterprise Office, St George’s University of London, Cranmer Terrace, London, SW17 0RE UK; 10 Department of Clinical Physiology and Nuclear Medicine, Rigshospitalet, Nordre Ringvej 57, DK-2600 Glostrup, Denmark; 11 Robertson Centre for Biostatistics, University of Glasgow, Glasgow, G12 8QQ UK; 12 Cerebrovascular Disease, St George’s University of London, Cranmer Terrace, London, SW17 0RE UK

**Keywords:** Tadalafil, Cerebral blood flow, Vascular cognitive impairment, Vascular dementia, Phosphodiesterase, MRI, Arterial spin labelling

## Abstract

**Background:**

Cerebral small vessel disease is a common cause of vascular cognitive impairment in older people, with no licensed treatment. Cerebral blood flow is reduced in small vessel disease. Tadalafil is a widely prescribed phosphodiesterase-5 inhibitor that increases blood flow in other vascular territories. The aim of this trial is to test the hypothesis that tadalafil increases cerebral blood flow in older people with small vessel disease.

**Methods/design:**

Perfusion by Arterial Spin labelling following Single dose Tadalafil In Small vessel disease (PASTIS) is a phase II randomised double-blind crossover trial. In two visits, 7-30 days apart, participants undergo arterial spin labelling to measure cerebral blood flow and a battery of cognitive tests, pre- and post-dosing with oral tadalafil (20 mg) or placebo. Sample size: 54 participants are required to detect a 15% increase in cerebral blood flow in subcortical white matter (p < 0.05, 90% power). Primary outcomes are cerebral blood flow in subcortical white matter and deep grey nuclei. Secondary outcomes are cortical grey matter cerebral blood flow and performance on cognitive tests (reaction time, information processing speed, digit span forwards and backwards, semantic fluency).

**Discussion:**

Recruitment started on 4th September 2015 and 36 participants have completed to date (19th April 2017). No serious adverse events have occurred. All participants have been recruited from one centre, St George’s University Hospitals NHS Foundation Trust.

**Trial registration:**

European Union Clinical Trials Register: EudraCT number 2015-001235-20. Registered on 13 May 2015.

**Electronic supplementary material:**

The online version of this article (doi:10.1186/s13063-017-1973-9) contains supplementary material, which is available to authorized users.

## Background

Cerebral small vessel disease (SVD) is a frequent cause of vascular cognitive impairment (VCI) in older adults [1–4]. There is currently no licensed treatment for SVD or for VCI [1, 2]. There is evidence derived from some previous studies to suggest that cerebral blood flow (CBF) is reduced in SVD, particularly in subcortical white matter [5–10]. We hypothesised that increasing CBF has the potential to be both a symptomatic and a disease-modifying treatment for SVD and VCI.

Phosphodiesterase type 5 inhibitors (PDE5i) such as sildenafil and tadalafil are well-established pharmacological vasodilators that cause enhanced nitric oxide-cyclic guanosine monophosphate signalling in peripheral small arteries [11–13]. PDE5i are widely used in treatment of erectile dysfunction and pulmonary hypertension [13]. PDE5 messenger RNA and protein are also found in human brain tissue [12, 14, 15]. Side-effect profiles of PDE5i are well-known, and the drugs are well-tolerated in the target population [16–18]. In a meta-analysis of 28 placebo-controlled trials [18], the overall incidence of myocardial infarction, cardiovascular death or cerebrovascular death in tadalafil-treated patients did not differ from placebo. The incidence of these adverse events was independent of dosing regimen and duration of tadalafil therapy (up to 27 months) [18]. The choice of tadalafil (over other PDE5i) was based on long plasma half-life (17 h in healthy adults) [16, 17] and established brain penetration (brain-to-plasma ratio 1:10 in rodents and primates) [12, 19]. In this study, we will test whether single-dose tadalafil increases CBF in older people with neuroradiological and clinical evidence of SVD.

## Methods/design

### Objectives

The aim of this study is to test the hypothesis that tadalafil increases cerebral blood flow in subcortical areas in older people with symptomatic SVD.

### Study design

**Fig. 1:**
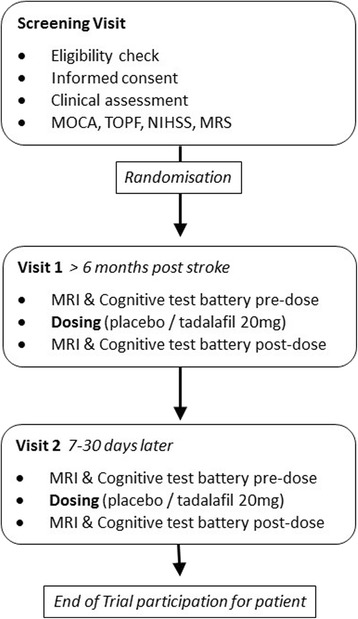
*MOCA* Montreal Cognitive Assessment, *TOPF* Test of Premorbid Functioning, *NIHSS* National Institutes of Health Stroke Scale, *MRS* Modified Rankin Scale, *MRI* Magnetic resonance imaging

Perfusion by Arterial Spin labelling following Single dose Tadalafil In Small vessel disease (PASTIS) is a phase II double-blind crossover trial. Participants are randomised to order of treatment (tadalafil 20 mg, placebo; oral administration). Two visits are performed 7–30 days apart, with perfusion magnetic resonance imaging (MRI) and a battery of cognitive tests performed before and 3–5 h after dosing (*see* Fig. 1).

**Fig. 2:**
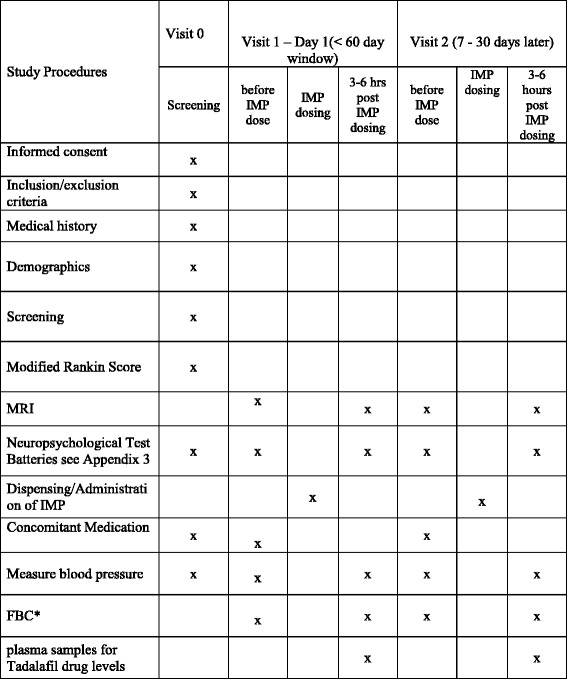
Standard Protocol Items: Recommendations for Interventional Trials (SPIRIT) checklist: schedule of enrolment, interventions and assessments in the Perfusion by Arterial Spin labelling following Single dose Tadalafil In Small vessel disease (PASTIS) trial. From PASTIS protocol version 4, 27 Jan 2016

A Standard Protocol Items: Recommendations for Interventional Trials (SPIRIT) checklist is shown in Fig. 2 (see also Additional file 1).

### Trial endpoints

The primary endpoints are change in regional CBF in two sub-cortical brain areas (deep white matter and deep grey nuclei). The secondary endpoints are (1) change in regional CBF in cortical grey matter, (2) change in neuropsychological test performance and (3) plasma tadalafil concentration dependence of any changes observed.

### Study setting

Participants are recruited from St George’s University Hospital NHS Foundation Trust and local Participant Identification Centre sites. All patient visits, data management and trial coordination are performed at St George’s. PASTIS has been adopted into the UK National Institute for Health Research Clinical Research Network Portfolio.

### Participant characteristics

Participants are older people (men and women) without a diagnosis of dementia who have radiological and clinical evidence of symptomatic SVD. After informed consent is obtained, the following activities will occur at a screening visit (*see* Fig. 1):

1. Trial eligibility criteria check
2. Medical history
3. Concomitant medication checklist: medications, doses and frequencies
4. MRI suitability/contraindication checklist
5. Participant demographics, including ethnic origin
6. Next-of-kin and general practitioner contact details to be recorded if not already in medical notes, or check if still current and up-to-date
7. Affix Clinical Trials Alert sticker to front of the medical notes
8. Complete case report form screening page, ensuring the participant’s trial identifier is included
9. Test of Premorbid Functioning (TOPF) to establish estimated levels of cognitive functioning pre-illness
10. National Institutes of Health Stroke Scale (NIHSS)
11. Montreal Cognitive Assessment (MoCA) to establish estimated levels of cognitive functioning
12. Record the modified Rankin Scale score (mRS)

#### Inclusion criteria

1. Radiological evidence of cerebral SVD, defined as MRI evidence of lacunar infarcts (≤1.5 cm maximum diameter) and/or confluent deep white matter hyperintensities (WMH) (≥grade 2 on Fazekas scale)
2. Clinical evidence of SVD, including the following:
  - Lacunar stroke syndrome with symptoms lasting >24 h, occurring ≥6 months prior to visit 1; or
  - Transient ischaemic attack (TIA) lasting <24 h with limb weakness, hemi-sensory loss or dysarthria ≥6 months previously and with MRI diffusion-weighted imaging performed acutely showing lacunar infarction, or, if MRI is not performed within 10 days of TIA, lacunar infarct in an anatomically appropriate area
3. Age ≥50 years
4. Imaging of the carotid arteries with Doppler ultrasound, computed tomographic angiography or magnetic resonance angiography in the previous 12 months demonstrating <70% stenosis in both internal carotid arteries *or* <50% stenosis in both internal carotids if measured in previous 12–60 months

#### Exclusion criteria

1. Known diagnosis of dementia
2. Cortical infarct (>1.5 cm maximum diameter)
3. Systolic blood pressure (BP) <90 mmHg and/or diastolic BP <50 mmHg
4. Creatinine clearance <30 ml/minute
5. Severe hepatic impairment
6. History of lactose intolerance
7. Concomitant use of PDE5i (e.g., sildenafil, tadalafil, vardenafil)
8. Receiving nicorandil or nitrates (e.g., isosorbide mononitrate, glyceryl trinitrate)
9. Weight >130 kg
10. Uncontrolled cardiac failure
11. Persistent or paroxysmal atrial fibrillation
12. History of gastric ulceration
13. History of ‘sick sinus syndrome’ or other supraventricular cardiac conduction conditions
14. Uncontrolled chronic obstructive pulmonary disease
15. Stroke or TIA within 6 months
16. MRI not tolerated or contraindicated
17. Known monogenic causes of stroke (e.g., cerebral autosomal dominant arteriopathy with subcortical infarcts and leukoencephalopathy)
18. Unable to provide informed consent

### Randomisation

The randomisation list will be generated by Sharp Clinical Services (http://www.sharpservices.com/our-facilities/sharp-clinical-services-wales/; Crickhowell, UK) and will be done in blocks, as detailed in the client study information form kept in the sponsor site file. The participants will be acting as their own controls. Each participant will receive on two separate occasions a placebo dose and a tadalafil 20-mg immediate dose which appear identical in size, shape, weight and colour.

The patient pack numbers on the pharmacy shelf correlate directly with the next available pack number on the blinded randomisation list held in the pharmacy site file. Each patient pack contains two bottles, labelled as bottle A and bottle B. The randomisation list will be confidential to the trial statistician and will be summarised as treatment arm A and B, and not by tadalafil and placebo.

### Measurement of regional cerebral blood flow

Whole-brain perfusion will be determined by pseudo-continuous arterial spin labelling (ASL) [20] in a 3-T MRI scanner (Achieva TX MRI scanner, Philips Medical Systems, Eindhoven, Netherlands). A total of 20-minute pseudo-continuous ASL acquisition time will be used to provide an adequate signal-to-noise ratio for CBF quantification in white matter. Other image data acquired will be as follows: an M_0_ image, to enable quantification of CBF; high-resolution 3D T1-weighted images for identification of grey and white matter regions of interest (including deep grey matter structures) for ASL analysis [20] and to map the ASL data to a standard brain atlas; fluid-attenuated inversion recovery (FLAIR) for delineation of WMH; and susceptibility-weighted imaging for detection of micro-haemorrhages. These will provide participant-specific WMH load and location of WMH. Total scanning time is under 60 minutes per MRI session.

### Cognitive testing

Scores derived from the TOPF and MoCA instruments are recorded at the screening visit. These are included in the analyses as baseline data. They are not used as inclusion or exclusion criteria.

At the two dosing visits, the following neuropsychological tests are used: the Reaction Time subtest of the Cambridge Cognition Cambridge Neuropsychological Test Automated Battery, the Speed of Information Processing subtest of the Brain Injury Rehabilitation Trust Memory and Information Processing Battery, the Digit Span forwards and backwards subtest of the Repeatable Battery for the Assessment of Neuropsychological Status (RBANS), and the Semantic Fluency subtest of RBANS.

### Biochemical analyses

A blood sample is taken at the end of visits 1 and 2 for haematocrit and full blood count analysis. Plasma samples are stored at −80 °C for subsequent analysis of plasma tadalafil concentration.

### Details of the intervention

**Fig. 3:**
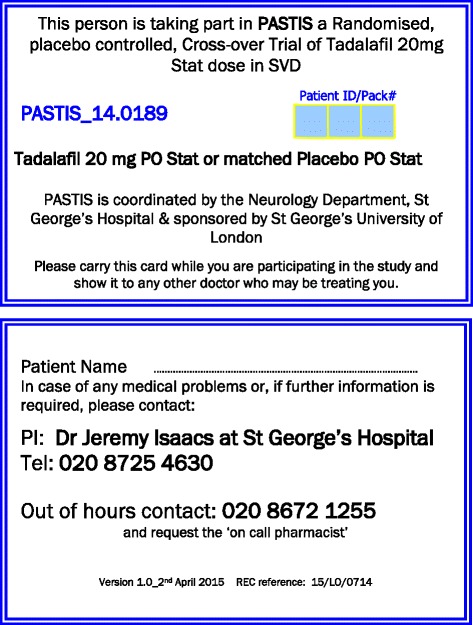
PASTIS trial emergency 24-hour contact card, supplied to each participant

Each participant pack contains one bottle containing a single tadalafil 20-mg capsule and one identical bottle containing a single matched placebo capsule. At each visit, participants undergo cognitive tests and the first MRI scanning session of the day. Participants are then observed to swallow the appropriate investigational medicinal product (IMP) capsule and receive a standard light lunch (450–750 kcal and 500 ml of fluid). They undergo an equivalent, parallel version of the cognitive tests and the second MRI session of the day 3–5 h later. All participants are given a 24-h emergency contact card with the study title, details of the IMP, their participant trial number, investigator’s contact details, and out-of-hours contact details (*see* Fig. 3).

All those involved in the study (researchers, radiologists, pharmacists and participants) are blinded to treatment allocation for the duration of the study. Emergency un-blinding will take place in circumstances such as serious adverse events (SAEs). Any SAEs and safety endpoints will be reported in line with clinical trial regulations (SI2004/1031) and the sponsor’s procedures. We do not anticipate any serious adverse reactions to the medication, because tadalafil is widely used clinically and is well-tolerated. The starting point for SAE monitoring is the first intervention visit, ending 5 days after the second visit (based on a drug elimination period of 6 half-lives for the study medication, using a 20-h half-life for tadalafil).

### Power calculation

On the basis of previous ASL studies of regional CBF, we estimate baseline perfusion of 30 (±10) ml/100 g/minute (mean ± SD) in subcortical white matter and 70 (±15) ml/100 g/minute in deep grey nuclei [21, 22]. To detect a treatment effect of 15% (mean paired difference) with statistical power of 90%, a sample size of 24 is required in deep grey matter nuclei and 54 in subcortical white matter. We aim to recruit a target cohort of 54.

### Statistical analysis

Baseline characteristics (age, sex, ethnic group, baseline BP, mRS score, NIHSS, TOPF, MoCA) will be summarised as mean (SD) or median (Q1, Q3) for continuous variables, depending on distribution, and as number (percent) for categorical variables. Changes in outcome variables will be calculated for each participant at each visit as post-dose value minus pre-dose value. Data will be analysed using a linear mixed effects regression model with fixed effects for treatment (drug vs. placebo), visit (visit 1, visit 2), treatment sequence and baseline response, as well as a random effect for participant nested within treatment sequence. Carry-over will be investigated by the treatment-by-visit interaction. If statistically significant, data from each visit will be analysed separately within linear regression models adjusted for treatment and pre-dose value. Clinical variables and other possible confounders (e.g., BP at the time of the scan) will be included in the linear mixed effects models as adjustment variables. These will be pre-specified in the statistical analysis plan.

All analyses will be done on an intention-to-treat basis, and no adjustment will be made for missing data. Statistical analyses will be performed using SAS® for Windows version 9.3 or later software (SAS Institute, Cary, NC, USA). A *p* value >0.05 indicates the absence of a statistically significant effect.

### Data monitoring

Monitoring is performed by the sponsor clinical trials monitor in accordance with an agreed risk-based monitoring plan. Case report form entries are verified against the source documents and the participants’ medical notes. All data are entered directly from case report forms to the PASTIS Microsoft Access (Microsoft Corp., Redmond, WA, USA) database by the PASTIS research team. Data transfer from the case report form will be double-checked, and where corrections are required, these will carry a full audit trail and justification. Trial data storage conforms to St George’s institutional information governance policies. Trial data, evidence of monitoring and system audits will be made available for inspection by the sponsor and regulatory authorities as required.

## Discussion

In this randomised, double-blind, crossover phase II study, we will test whether tadalafil (20 mg) increases CBF in older people with SVD. Tadalafil was chosen over other PDE5i (e.g., sildenafil or vardenafil) because of the documented brain penetration [12, 19] and longer plasma half-life of tadalafil [16, 17]. In the present trial, we are simply testing for acute changes in response to a single dose of tadalafil. For this purpose, a crossover design appeared optimal. In the event that a positive outcome is detected in the present study, it appears likely that a subsequent study testing tadalafil over a longer dosing period will be required. This will be needed to explore whether any tadalafil-mediated actions are maintained with chronic dosing and to test for any additional adverse reactions in participants who are likely to be taking concomitant stroke medications.

ASL was chosen to quantify regional CBF because it does not require injected radioisotopes or gadolinium compounds as tracers [20–22]. This MRI-based approach also enables acquisition of high-resolution 3D T1-weighted images, T2-weighted FLAIR images and susceptibility-weighted imaging. The neuropsychological tests that are used were chosen because each has four parallel versions of the test to be applied at each screening point (Fig. 1). The cognitive tests used measure processing speed, attention and executive function, which are affected in SVD, as well as working memory and semantic fluency. Nevertheless, it may be difficult to detect cognitive changes in such short-term follow-up as is employed in the present study. The cognitive data obtained from this trial may be of value in assessing sample size and feasibility for any subsequent trial of tadalafil in relation to cognitive function.

In addition to the European Union Clinical Trials Register (EudraCT number 2015-001235-20, date of registration 13 May 2015), the trial has been registered with ClinicalTrials.gov (NCT02450253, date of registration 18 May 2015). No SAEs have been observed so far. Inadvertent un-blinding due to the erectile effects of tadalafil has not occurred so far as we are aware. Spontaneous penile erection has been reported in a modest fraction (11%) of subjects taking 20 mg of tadalafil [16, 17]. PASTIS is the first phase II clinical trial of a selective PDE5i in older people with symptomatic SVD. Outcome data are expected in late 2017 and may inform a larger trial for re-purposing of tadalafil in SVD and VCI.

## Trial status

The trial commenced on 4th September 2015. The PASTIS trial is ongoing. Patient recruitment has not been completed. As of 19 April 2017, 36 participants have completed the protocol.

## Additional file

Additional file 1: SPIRIT 2013 checklist: recommended items to address in a clinical trial protocol and related documents. (DOC 120 kb)

## Abbreviations

ASL: Arterial spin labelling
BP: Blood pressure
CBF: Cerebral blood flow
FLAIR: Fluid-attenuated inversion recovery
IMP: Investigational medicinal product
MoCA: Montreal Cognitive Assessment
MRI: Magnetic resonance imaging
mRS: Modified Rankin Scale
NIHSS: National Institutes of Health Stroke Scale
PASTIS: Perfusion by Arterial Spin labelling following Single dose Tadalafil In Small vessel disease
PDE5i: Phosphodiesterase type 5 inhibitor
RBANS: Repeatable Battery for the Assessment of Neuropsychological Status
SAE: Serious adverse event
SPIRIT: Standard Protocol Items: Recommendations for Interventional Trials
SVD: Small vessel disease
TIA: Transient ischaemic attack
TOPF: Test of premorbid functioning
VCI: Vascular cognitive impairment
WMH: White matter hyperintensities
